# Laser-assisted synthesis of two-dimensional transition metal dichalcogenides: a mini review

**DOI:** 10.3389/fchem.2023.1195640

**Published:** 2023-04-25

**Authors:** Hanxin Wang, Manzhang Xu, Hongjia Ji, Tong He, Weiwei Li, Lu Zheng, Xuewen Wang

**Affiliations:** ^1^ Frontiers Science Center for Flexible Electronics (FSCFE), Institute of Flexible Electronics (IFE), Northwestern Polytechnical University, Xi’an, China; ^2^ MIIT Key Laboratory of Flexible Electronics (KLoFE), Northwestern Polytechnical University, Xi’an, China; ^3^ Shaanxi Key Laboratory of Flexible Electronics (KLoFE), Northwestern Polytechnical University, Xi’an, China; ^4^ Institute of Basic and Translational Medicine, Xi’an Medical University, Xi’an, China; ^5^ School of Chemistry and Chemical Engineering, Shaanxi Normal University, Xi’an, China; ^6^ Key Laboratory of Flexible Electronics of Zhejiang Province, Ningbo Institute of Northwestern Polytechnical University, Ningbo, China

**Keywords:** laser, two-dimensional materials, transition metal dichalcogenides, synthesis methods, mechanism

## Abstract

The atomically thin two-dimensional (2D) transition metal dichalcogenides (TMDCs) have attracted the researcher’s interest in the field of flexible electronics due to their high mobility, tunable bandgaps, and mechanical flexibility. As an emerging technique, laser-assisted direct writing has been used for the synthesis of TMDCs due to its extremely high preparation accuracy, rich light–matter interaction mechanism, dynamic properties, fast preparation speed, and minimal thermal effects. Currently, this technology has been focused on the synthesis of 2D graphene, while there are few literatures that summarize the progress in direct laser writing technology in the synthesis of 2D TMDCs. Therefore, in this mini-review, the synthetic strategies of applying laser to the fabrication of 2D TMDCs have been briefly summarized and discussed, which are divided into top-down and bottom-up methods. The detailed fabrication steps, main characteristics, and mechanism of both methods are discussed. Finally, prospects and further opportunities in the booming field of laser-assisted synthesis of 2D TMDCs are addressed.

## 1 Introduction

Flexible electronics is a disruptive science and technology based on a high degree of interdisciplinary integration, which provides novel opportunities for the development of the next generation of the information technology revolution and the era of intelligent manufacturing (Zheng et al., 2022; Li et al., 2023). The two-dimensional materials, especially transition metal dichalcogenides have become trending research topics of flexible electronics due to their unique structure, mechanical flexibility, tunable bandgaps, and high mobility (Mak et al., 2010; Fan et al., 2015; Kim et al., 2015; Leong, 2020; Yu et al., 2020; Liu et al., 2021; Wu et al., 2022).

Currently, significant research has been focused on the efficient synthesis of high-quality large-scale 2D TMDCs with simple crafts and low costs, owing to the prerequisite and key factor for their diverse advanced industrial application (Lee et al., 2012; Lei et al., 2013; Mahjouri Samani et al., 2014; Shim et al., 2014; Xia et al., 2014; Xu et al., 2021b). Various synthesis techniques, such as chemical vapor deposition (CVD), metal–organic chemical vapor deposition (MOCVD), and molecular beam epitaxy (MBE), have been used to control the growth of 2D TMDCs on different substrates and show great potential in growing high-quality crystalline 2D TMDCs under high temperature (Yu et al., 2017; Gao et al., 2021). However, patterning TMDCs cannot be achieved in the synthesis process, and an additional patterning process is still required during the device fabrication (Mak et al., 2010). In recent years, direct laser writing technology has been widely used in the synthesis of materials because of the ability to minimize thermal effects, rich light–matter interaction mechanism, kinetic properties, the high fabrication accuracy, and fast preparation speed (Lu et al., 2014; Jung et al., 2019; Park et al., 2020a; Park et al., 2020b; Hu et al., 2020). In addition, maskless and lithography-free properties of laser patterning can enable one-step fabrication of the desired specific patterns, avoiding material contamination while reducing the process flow (Cao et al., 2013; Mohapatra et al., 2020). All preparation methods of synthesizing 2D TMDCs and their advantages and disadvantages are shown in Table 1, which shows the salient features of direct laser writing technology from multiple perspectives.

**TABLE 1:** Summary of preparation methods of 2D TMDCs.

Method	Advantage	Disadvantage
Mechanical exfoliation	High quality, simple	Low yield, challenging to mass production
Liquid-phase exfoliation	High preparation efficiency, simple	Small lateral size, low yield
Physical vapor deposition	High quality, low cost	Low repetition rate, complex process
Atomic layer deposition	High repetition rate	High cost, complex process
Hydrothermal	High yield, low cost	Low controllability, unstable grain size, low quality
CVD	Low cost, high quality, possible to mass production	High preparation temperature, low repetition rate
MOCVD	High deposition rate, high quality	Chemical pollution, high cost, long production cycle
MBE	Atomic-level control, high purity	Rigorous conditions, expensive equipment
Laser-assisted synthesis	Low cost, high repetition rate, patternable	Hardly achieve nanometer-level resolution, numerous defects

In this mini-review, we systematically summarize the latest studies on the laser-assisted synthesis of 2D TMDCs. Basically, there are essentially two classes of synthetic strategies: top-down and bottom-up methods. With regard to top-down methods, the advantages and mechanisms of the laser–material interactions are discussed, including laser exfoliation, laser thinning, and laser-driven phase transition. With regard to bottom-up methods, the growth mechanisms are discussed, including direct laser writing, laser heterostructures, and laser doping. Finally, prospects and further opportunities in the laser-assisted synthesis of 2D TMDCs are also addressed.

## 2 Top-down synthesis methods

Top-down synthesis methods generally require synthesizing the 2D TMDC crystal first, followed by further processing via laser–material interactions to improve the morphology or properties. Specifically, it can be classified into laser stripping, laser thinning, and laser-driven phase transition.

### 2.1 Laser exfoliation

The unique characteristics of the laser, such as ultra-high-peak power density and short pulse duration, far exceed conventional heating methods, such as electric or gas heating, thus making it possible to exfoliate monolayers of two-dimensional materials. The van der Waals interaction between the layers of TMDC materials can be broken by the laser, which enables the efficient and controllable preparation of TMDCs with a specific number of layers in a relatively short time. In addition, this technique has now been used to exfoliate a variety of 2D TMDC materials such as MoS_2_, MoSe_2_, and WS_2_ under different conditions (Schuffenhauer et al., 2005; Hu et al., 2006; An et al., 2018; Gao et al., 2019; Zhai et al., 2021; Zuo et al., 2021; Kimiagar and Abrinaei, 2023).

**FIGURE 1:**
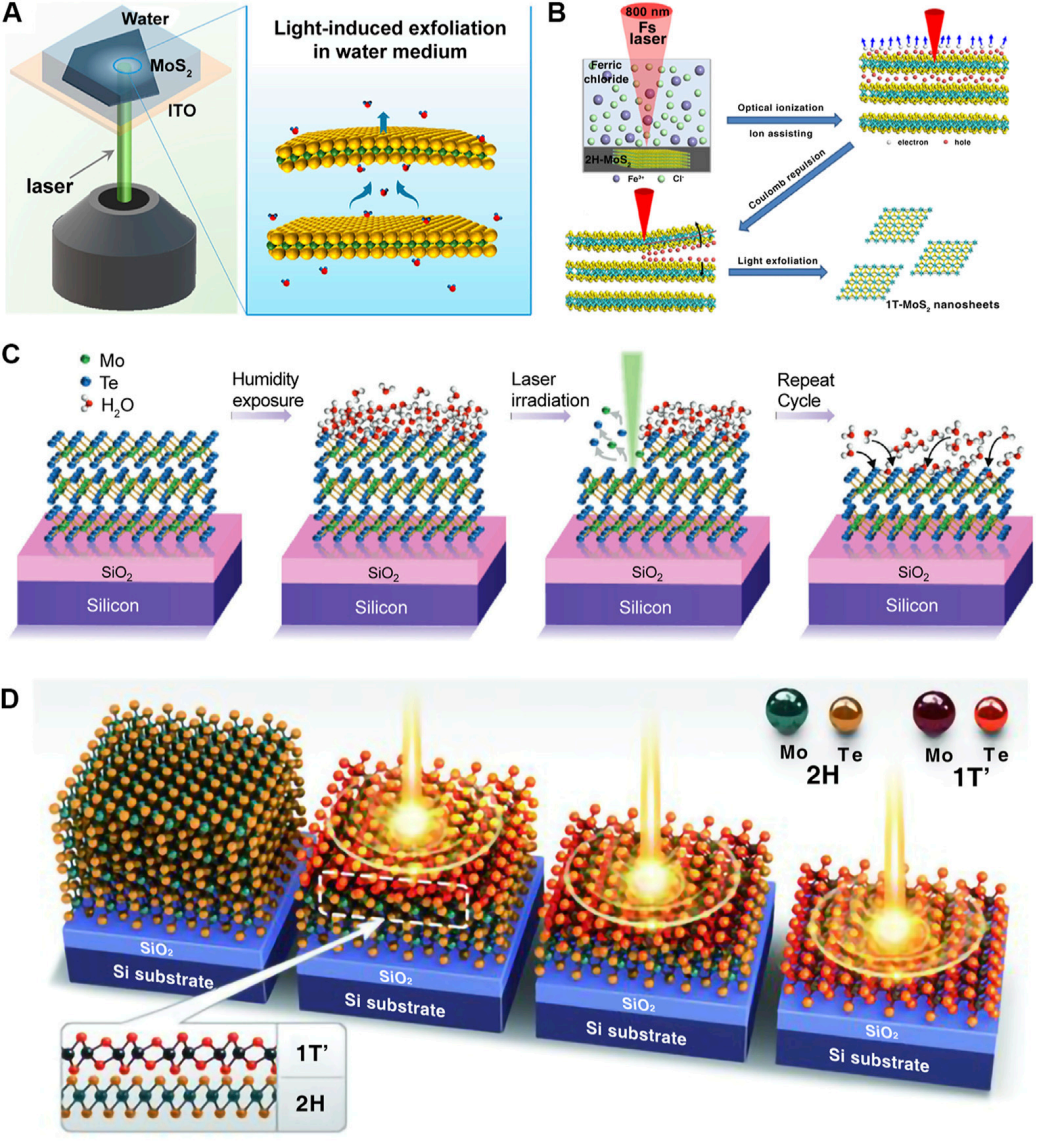
Top-down synthesis methods. **(A)** Schematic of the laser exfoliation device for 2H-MoS_2_ flakes in aqueous media (Zhai et al., 2021) (Copyright 2021, American Chemical Society). **(B)** Schematic of the preparation of MoS_2_ nanosheets through femtosecond laser exfoliation (Zuo et al., 2021) (Copyright 2021, American Chemical Society). **(C)** Schematic of the mechanism for laser thinning of MoTe_2_ (Nagareddy et al., 2018) (Copyright 2018, Wiley-VCH). **(D)** Schematic of the laser-driven phase patterning process of MoTe_2_ from 2H to 1T′ (Cho et al., 2015) (Copyright 2015, American Association for the Advancement of Science).

TMDCs are layered materials that form an MX_2_ crystal structure composed of strongly covalently bonded X-M-X sandwiches, and the X-M-X layers are held together by weak van der Waals forces (An et al., 2018). Zhai et al. (2021) created a method for exfoliating 2H-phase MoS_2_ (2H-MoS_2_), that is, both efficient and free from contaminants, and also the exfoliation process can be directly observed by optical microscopy (Figure 1A). Compared to other exfoliation techniques, this method is significantly faster and easier. The thickness of the irradiated region underwent a reduction approximately from 100 nm to 5 nm, and the area of the peeled region is also much larger than the diameter of the laser spot used. They believed that the light illumination and water medium are essential for the laser exfoliation of 2D TMDCs. When 2H-MoS_2_ layers are exposed to laser irradiation, the rapid vaporization of water molecules between the layers results in the exfoliation of MoS_2_ sheets, and the exfoliation process can be controlled by applying a bias voltage. The related research results indicated that water molecules could be incorporated into the interlayer spaces of 2H-MoS_2_ sheets (Levita and Righi, 2017; Ma et al., 2018). The phase transition may accompany the exfoliation process. Gao et al. (2019) achieved a one-step exfoliating bulk 2H-MoS_2_ into 2H and 1T MoS_2_ nanosheets using pulsed laser irradiation. Figure 1B shows a conceptual model of this experimental mechanism, suggesting that the 2H-phase MoS_2_ can be reversed into 1T MoS_2_ with the assistance of the chemically doped Fe^3+^ ions. In addition, the capability of the protic solvent also provides the proton to the reaction environment and plays a crucial role in triggering the phase transition.

### 2.2 Laser thinning

Laser thinning techniques have provided a direct and site-specific method for removing layers and obtaining “on-demand” 2D TMDCs (Castellanos Gomez et al., 2012; Lu et al., 2014; Sunamura et al., 2016; Kim et al., 2017; Park et al., 2017; Gong et al., 2018; Nagareddy et al., 2018; Rho et al., 2019; Tran-Khac et al., 2019; Wang et al., 2020; Kang et al., 2021). Unlike the traditional gas or plasma etching thinning method, laser thinning shows high accuracy on thinned layers, flexible programmable patterning mode, and high thinning efficiency. Nagareddy et al. (2018) demonstrated an efficient laser thinning method, which was constructed by the Raman spectroscopy and coupled with an atomic force microscope. The experimental setup was placed in an airtight environment with strictly controlled humidity. Using ultra-low laser power, the thickness of MoTe_2_ film can be controlled layer by layer from multilayer to monolayer, as shown in Figure 1C. In addition, the thickness reduction shows the linear correlation between the thickness reduction and the number of scans. The laser thinning process is achieved through the sublimation of the top layer, mainly caused by the conversion of light absorbed by the material into heat energy (Lu et al., 2014). The heat generated by the conversion of absorbed laser into energy is difficult to dissipate through the substrate due to the poor coupling between TMDC thin layers that are mediated by van der Waals forces (Su et al., 2021). Thus, until the laser power is increased, the bottom layer remains in close contact with the SiO_2_/Si substrate, acting as a heat sink to prevent removal.

### 2.3 Laser driven phase

The exploration and manipulation of a novel phase of matter is a primary pursuit for materials research. The emergence of atomically 2D TMDCs has enabled the examination of diffusive, displacive, and quantum phase transitions. Cho et al. (2015) demonstrated a novel method of localized polymorph engineering and realized the laser-induced phase transition of MoTe_2_. The multilayer 2H-MoTe_2_ flake with about 30 layers was obtained by mechanical exfoliation. The schematic diagram of the phase transition mechanism is illustrated in Figure 1D. Under laser irradiation, the thickness of the irradiated region in 2H-MoTe_2_ decreased by a few layers and transformed to 1T′ phase. During the irradiation process, the 1T′ MoTe_2_ layer remained due to the heat sink effect of the SiO_2_ substrate. It should be noticed that the phase transition is irreversible from 1T′ to 2H phase even under the higher energy or intensity of the laser. The driving force for the one-way phase transition is caused by the irreversible Te vacancy created under laser irradiation. Compared with conventional methods such as heat treatment, strain engineering, charge transfer, and plasma irradiation, laser-induced phase transition engineering allows for specific phase transition sites and patterned processing without impurities during the phase transition (Chen Z. R. et al., 2019).

## 3 Bottom-up synthesis methods

Unlike the top-down synthesis method, the bottom-up synthesis method is more efficient and cost-effective in the synthesis of 2D TMDC materials because it does not require the preparation of precursors using other methods. With the assistance of a laser, large-area patterned TMDCs, heterostructure, and atom doping can be realized.

### 3.1 Laser directed synthesis

**FIGURE 2:**
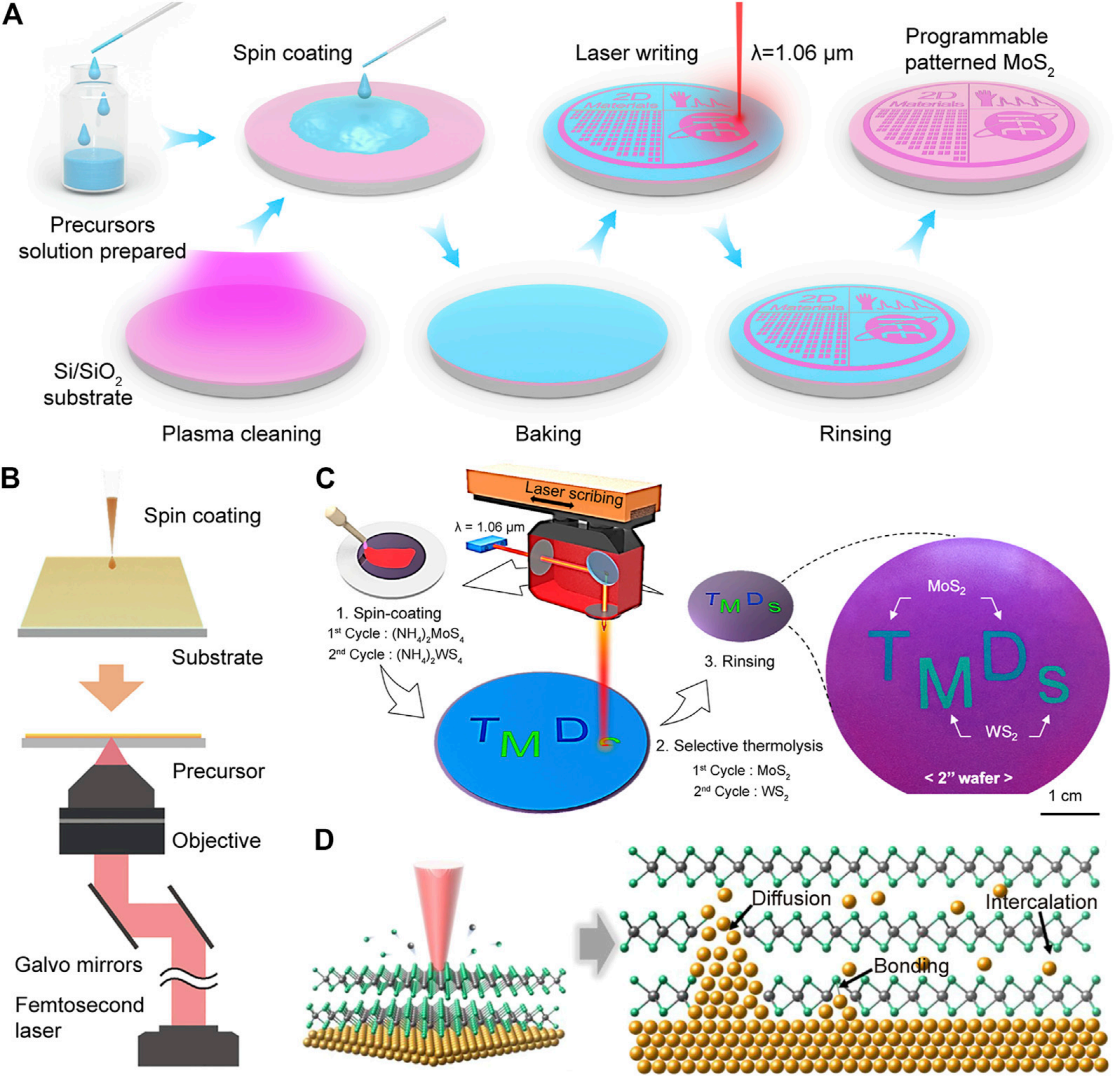
Bottom-up synthesis methods. **(A)** Flow diagram of direct laser writing, a method for the laser-directed synthesis of MoS_2_ on the SiO_2_/Si wafer (Xu et al., 2021a) (Copyright 2021, Elsevier). **(B)** Schematic of MoS_2_ synthesized by femtosecond laser (Xu et al., 2022) (Copyright 2022, American Chemical Society). **(C)** Schematic of the layer synthesis of MoS_2_–WS_2_ heterostructure (Park et al., 2020a) (Copyright 2020, American Chemical Society). **(D)** Schematic of Au-doped MoS_2_ (Huo et al., 2021) (Copyright 2021, American Chemical Society).

Direct laser writing technology can directly pattern 2D TMDC materials without mask and lithography, reducing the process flow of device fabrication and the risk of chemical contamination. Generally, the fiber laser (1.06 *μ*m), carbon dioxide laser (10.6 *μ*m), and femtosecond laser (780 nm) have been adopted for the synthesis of 2D TMDCs (Hu et al., 2018). Xu et al. (2021a) reported a method for the efficient synthesis of wafer-scale MoS_2_ using a direct laser writing technique, and the workflow is shown in Figure 2A. With heating by a 1.06 *μ*m commercial fiber laser, the MoS_2_ can be programmable with text, lines, patterns, and matrices in a few minutes. The laser interacts with the precursor, and the reaction temperature facilitates the thermal decomposition of (NH_4_)_2_MoS_4_ into MoS_2_. Apart from the thermal decomposition, the photochemical reaction induced by two-photon absorption can also be adopted for synthesizing 2D TMDCs. As shown in Figure 2B, the femtosecond direct laser writing was carried out to synthesize MoS_2_ under an objective lens. Molybdenum acetylacetonate and carbon disulfide were adopted as the molybdenum and sulfur sources. The two-photon absorption occurs in the focal point, synthesizing MoS_2_ from the molybdenum metal complex via a photochemical reaction. With the assistance of a femtosecond laser, a minimal line width of approximately 780 nm can be achieved.

### 3.2 Laser heterostructure

The direct laser writing technique allows the fabrication of single 2D TMDCs and the direct preparation of 2D TMDC heterostructures. Compared with the transfer method, the laser heterostructure process can be programmable patterned, avoiding material damage and pollution (Li et al., 2020). In addition, the synthesis efficiency of heterostructures is greatly improved compared with CVD. Park et al. (2020a) successfully prepared WS_2_–MoS_2_ heterostructures vertically by two-step laser scribing, as shown in Figure 2C. The MoS_2_ layer was first synthesized by the thermal decomposition of (NH_4_)_2_MoS_4_ when the temperature increased over 700°C under laser irradiation. While for the heterostructures, due to the different optical absorption coefficients, the MoS_2_ layer shows a minor temperature increase, and only the (NH_4_)_2_WS_4_ layer absorbed the laser energy and selectively decomposed into WS_2_. Thus, the selective growth process by laser effectively produces layer-by-layer 2D TMDC heterostructures in a programmable pattern.

### 3.3 Laser doping

The impurity atom can modulate the physical and chemical properties of semiconductors such as electrical and optical. A small amount of elemental doping could affect the energy bands enhancing semiconductor conductivity. However, doping elements in 2D TMDCs are relatively difficult and generate more defects in the 2D TMDCs. In addition, the weak interaction will result in unstable interfaces due to the interaction between doped elements and 2D TMDCs (Mak et al., 2010). Laser provides a simple way for 2D TMDC doping, which is promising for future electronic applications (Chen J. et al., 2019). One way to achieve laser doping in 2D TMDCs is by dissolving the doping substance in the precursor, which results in doping during formation (Hu et al., 2020). In addition, doping of elements in preprepared 2D TMDCs can also be accomplished by introducing gas molecules or solids containing the desired doping atoms (Afaneh et al., 2018; Rho et al., 2019; Huo et al., 2021). For example, Huo et al. (2021) reported an Au-doped MoS_2_ process by doping a solid source, as shown in Figure 2D. MoS_2_ was transferred to the Au electrode, and with femtosecond laser irradiation, the Au atom can be intercalated and diffused into MoS_2_. The interface diffusion and chemical bonding of Au reduced the Schottky barrier of the metal–semiconductor interfaces, which could enhance the performance of the devices.

## 4 Conclusion and outlook

This mini-review summarized the top-down and bottom-up methods for the laser synthesis of 2D TMDCs. Compared with the traditional methods, the laser synthesis methods have the advantages of flexibility, convenience, efficiency, and controllability. Laser synthesis technology can directly produce large-area, high-quality TMDCs, alloys, and heterostructure, which is a promising sign for future industrial-scale manufacturing.

Laser synthesis of 2D TMDCs is an emerging field, and some issues need to be further addressed, especially for the laser–reactive matter interaction. In addition, the synthesis of sulfides in the atmospheric environment is the focus of attention, while the atmosphere protection and hydrogen participation would be focused while synthesizing selenide and telluride. In addition, recent works on laser synthesis of 2D TMDCs constructed on the rigid substrate (such as SiO_2_/Si) or liquid environments and direct laser synthesis on flexible substrates (such as polyimide, polydimethylsiloxane, polyethylene terephthalate, and polyethylene naphthalene) have not been realized yet, which would offer another chance for realizing applications in flexible electronics.
